# Gas Leak Location Detection Based on Data Fusion with Time Difference of Arrival and Energy Decay Using an Ultrasonic Sensor Array

**DOI:** 10.3390/s18092985

**Published:** 2018-09-07

**Authors:** Tao Wang, Xiaoran Wang, Mingyu Hong

**Affiliations:** School of Automation, Beijing Institute of Technology, No.5 South Zhongguancun Street, Haidian District, Beijing 100081, China; beifen_wxr@163.com (X.W.); hongmingyu2011@126.com (M.H.)

**Keywords:** gas leak location, data fusion, TDOA, ED, ultrasonic sensor array

## Abstract

Ultrasonic gas leak location technology is based on the detection of ultrasonic waves generated by the ejection of pressured gas from leak holes in sealed containers or pipes. To obtain more accurate leak location information and determine the locations of leak holes in three-dimensional space, this paper proposes an ultrasonic leak location approach based on multi-algorithm data fusion. With the help of a planar ultrasonic sensor array, the eigenvectors of two individual algorithms, i.e., the arrival distance difference, as determined from the time difference of arrival (TDOA) location algorithm, and the ratio of arrival distances from the energy decay (ED) location algorithm, are extracted and fused to calculate the three-dimensional coordinates of leak holes. The fusion is based on an extended Kalman filter, in which the results of the individual algorithms are seen as observation values. The final system state matrix is composed of distances between the measured leak hole and the sensors. Our experiments show that, under the condition in which the pressure in the measured container is 100 kPa, and the leak hole–sensor distance is 800 mm, the maximum error of the calculated results based on the data fusion location algorithm is less than 20 mm, and the combined accuracy is better than those of the individual location algorithms.

## 1. Introduction

In recent years, as the scale of industry has gradually expanded, pressure vessels and pipes have become increasingly used. However, sealed structures are easily damaged by various natural or man-made factors such as corrosion, abrasion and shipping accidents. The consequences of gas leaks include the loss of materials and energy, fire and explosion hazards, environmental pollution and decreased production efficiency [1,2,3,4]. Therefore, it is vital to ensure the tightness of pressure vessels before and during their use; doing so requires a leak location approach to identify any leak holes so that the dangers associated with leakage accidents can be avoided [5].

Widely used leak detection methods include differential pressure methods [6,7,8], mass spectrometry methods [9], infrared thermal imaging methods [10] and ultrasonic methods [11]. The features of these methods are listed in Table 1.

Among them, the accuracy of ultrasonic leak detection methods is higher than that of differential pressure methods, and the manufacturing costs are lower than those of infrared thermal imaging methods and mass spectrometry methods, the operation and devices are also simpler, therefore, ultrasonic methods are suitable for large-scale application.

Ultrasonic gas leak detection technology analyzes ultrasonic waves generated by the ejection of high-pressure gas from leaking holes in sealed containers or pipes to determine the presence or absence of leaks, the location of any leaks and the size of the leaking holes. That is, the location of a leak point is estimated by calculating the position of the ultrasonic sound source. Essentially, the location of a leak point is inferred on the basis of the location of the sound source.

As early as 1995, Brandstein conducted in-depth research regarding sound source location technology at the theoretical level and introduced the principles and implementation processes of various sound source location methods [12]. Silverman et al. began to use sensor arrays for sound source location research in 1997, applying it to estimate and track the position of talking individuals [13]. In 1999, Reed et al. studied the propagation characteristics of signals and attempted to estimate the position of sound sources by using time arrival differences and signal propagation characteristics, and have illustrated the dependence of iterative algorithm results on the initial values [14].

In the field of applying sound source location technology for gas leak detection, in 2015, leak location based on an ultrasonic sensor array with the time difference of arrival (TDOA) location algorithm was studied by Yu [15]. In 2018, Bolotina et al. proposed a passive leak detection method for underwater pipelines by means of phased antenna arrays [16]. That same year, a ring sensor array is used to locate leaks in circular containers and pipes [17]. None of these ultrasonic leak location methods are yet mature because of limitations imposed by the use of two-dimensional planes, the working environment and/or the characteristics of the measured items. To address these shortcomings, in this paper we propose a leak location method based on fusing the TDOA location algorithm [18] and the energy decay (ED) location algorithm [19], in which we use a planar sensor array to estimate the location of leak holes at any position in three-dimensional space.

## 2. Principles of Ultrasonic Leak Detection

### 2.1. Generation of Ultrasonic Waves

**Figure 1:**
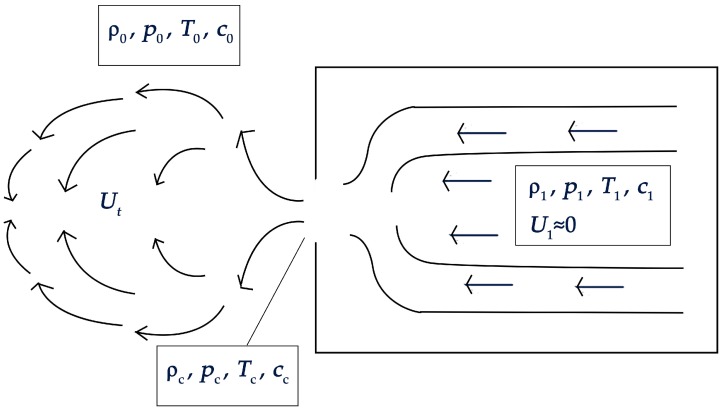
Gas leak model.

Owing to poor processing quality, unreasonable installation and/or their continuous use for long periods of time, fine pores and cracks can be caused on a variety of hydraulic and pneumatically sealed vessels, pipelines or welding processing interfaces. Meanwhile, leakages can occur because of the action of pressure systems, such as the compression of gas. When the pore size is sufficiently small, and the pressure difference between the inside and outside of the vessel is sufficiently large, the flow rate of the gas generated from the leakage of the pores is large, and the Reynolds number of the leaked gas is usually relatively high, so that a turbulent ejection is generated. In such turbulent ejections, vortices are generated from the initial section near the slit and propagate far away; these vortices constantly develop and break up, thus generating new vortices. The gas leakage model is shown in Figure 1. Regarding these eddies, Lighthill, in 1952, provided an explanation by showing that those vortices are actually the ‘sound’ of fluids [20].

In Figure 1, *ρ*_1_, *ρ*_c_ and *ρ*_0_ are the gas densities, *p*_1_, *p*_c_ and *p*_0_ are the gas pressures, *T*_1_, *T*_c_ and *T*_0_ are the absolute gas temperatures, *c*_1_, *c*_c_ and *c*_0_ are the sound speeds, and *U*_1_ and *U*_t_ are the gas flow rates. Among them, *ρ*_1_, *p*_1_, *T*_1_, *c*_1_ and *U*_1_ are the gas parameters in the container. It is assumed that the container is adiabatic, and the internal air flow velocity is zero; hence these parameters are set as stagnation parameters. *ρ*_c_, *p*_c_, *T*_c_, *c*_c_ and *U*_t_ are the air flow parameters at the outlet of the leak, *ρ*_0_, *p*_0_, *T*_0_ and *c*_0_ are the atmospheric gas parameters, and *p*_1_ and *p*_0_ are the absolute pressures. It is assumed that the interior of the container is isothermal with the ambient air, i.e., *T*_1_ = *T*_0_.

Usually, any energy losses caused by heat exchange between the ambient atmosphere and the ejected gas are negligible compared to the high-speed flow when the gas is ejected from the leak hole outward. The ejection of the gas may be considered as an isentropic flow. Therefore, the Reynolds number formula for the gas leak is:

(1) $$R_{e}=\frac{\rho_{c}U_{t}d}{\mu }\;$$

where *μ* is the viscosity of the gas at the outlet of the leak. At a temperature of 20 °C and a pressure of 101.325 kPa, the air viscosity is approximately 1.181 × 10^−5^ Pa·s. Finally, *d* is the leak hole diameter.

Gas leak ejections are commonly classified as blocked and non-blocked injections. Among them, for a non-blocked ejection, the absolute pressure inside the container and of the ambient atmosphere is compatible with the condition: $1<p_{1}/p_{0}<1.893$. At this time, the ejection is subsonic. The airflow speed is slower than the local speed of sound, and it increases when the pressure difference between the inside and outside of the detected vessel increases. When the ejection is blocked, the system is compatible with the condition: $p_{1}/p_{0}\geq 1.893$. The ejection at this time is sonic, and the airflow velocity is constant and equal to the local sound velocity, regardless of the pressure difference.

In non-blocked ejections, the flow outside the leak hole propagates at the speed of sound to the leak hole, so that the pressure at the outlet of the leak is equal to that of the ambient atmosphere. The gas parameters inside the container and at the outlet of the leak are substituted into the gas motion equation. According to the flow velocity and density at the leak hole in non-blocked and blocked ejections, the Reynolds number of the gas flow at the leak outlet is:

(2) $$R_{e}=\{\begin{array}{ll} \frac{d\frac{p_{1}}{RT_{0}}{\left(\frac{p_{0}}{p_{1}}\right)}^{\frac{1}{k}}\sqrt{\frac{2k}{k-1}RT_{0}\left[1-{\left(\frac{p_{0}}{p_{1}}\right)}^{\frac{k-1}{k}}\right]}}{\mu } & ,1<\frac{p_{1}}{p_{0}}<1.893\\ \frac{0.7607d\frac{p_{1}}{RT_{0}}\sqrt{kRT_{0}}}{\mu } & ,\frac{p_{1}}{p_{0}}\geq 1.893 \end{array}\;$$

where *R* is the gas constant that characterizes the relationship between the ideal gas temperature and energy. For air, *R* is 287.1 J/(kg·K). *k* is the specific heat ratio of the gas, where *k* = 1.40 here.

The frequency band of the ejection noise is very wide, i.e., approximately several hundred kilohertz [21]. After an in-depth experimental analysis, Ma Dayou et al. have provided an empirical formula for the peak frequency of the ejection noise, *f*_p_, as [22]:

(3) $$f_{p}=\frac{U_{t}}{5d}\;$$

**Figure 2:**
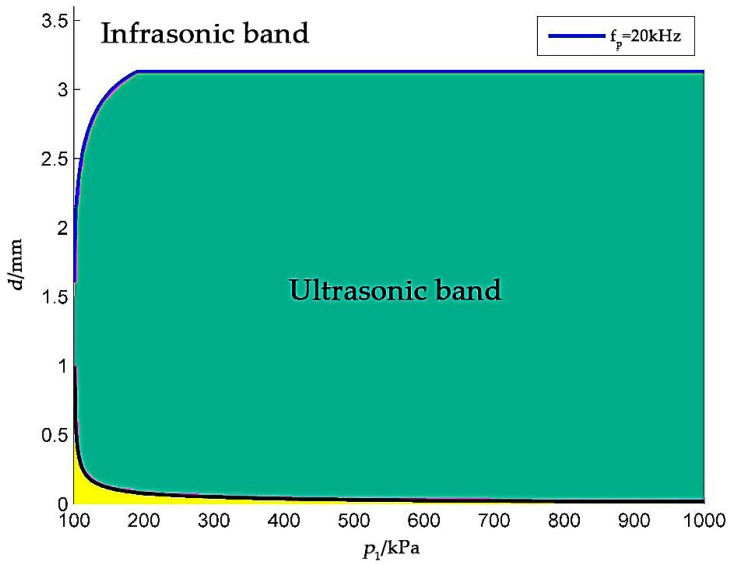
Peak frequency of the ejection waves.

According to Equations (2) and (3), a plot of the relationships among the frequency band at which the peak frequency of the leak hole noise is located, the leakage aperture and the absolute pressure within the detected part, is shown in Figure 2.

In Figure 2, the green region represents any ejection leakage that has a peak frequency higher than 20 kHz and that generates ultrasonic waves. The yellow area represents any non-turbulent ejection, in which it is believed that no noise is generated. Owing to the small leak hole aperture and the presence of a certain internal and external pressure difference, the frequency of the sound waves generated by vortices is generally higher than 20 kHz. Such ultrasonic waves that cannot be perceived by the human ear, but they can be detected by ultrasonic sensors, and after certain data processing and calculation steps, gas leak locations can be determined.

### 2.2. Decay Characteristics of Ultrasonic Waves

The ED location algorithm is based on the decay characteristics of ultrasonic waves [23,24]. When sound propagates in a homogenous medium, beam divergence and absorption occur, and when it propagates in an inhomogeneous medium, scattering also occurs. Dispersion refers to the expansion of the wavefront during the propagation of sound waves and the reduction of acoustic energy per unit area. Media absorption refers to the irreversible transformation of sound energy into heat energy and internal energy of the medium during the propagation process, thus resulting in a decay of energy. The usual source of leakage in engineering is pure air, which is a homogenous medium; hence we consider only divergence and absorption. Moreover, compared with that due to dispersion, the energy loss due to absorption is very small and can be neglected. After ignoring the absorption characteristics of the medium, the total acoustic power of the sound wave in the propagation process remains unchanged. The relationship between sound intensity, sound pressure and sound power is as follows:

(4) $$I=\frac{W}{S}=p^{2}/(\rho c)\;$$

where *I* is the sound intensity, *W* is the sound power, and *S* is the area of the acoustic wavefront, *p* is the sound pressure, *ρ* is the density of the propagation medium, and c is the speed of sound.

In practice, leak holes in vessels or pipes are so small that their pore sizes are usually on the order of 0.1 mm or even smaller. According to the frequency range of the waves produced by leakage, the wavelength range of them is between 1000 mm and 10 mm, which is much larger than the leak hole. When sound waves propagate in homogeneous medium the velocity and frequency stay constant, and the wave length is unchanged. Therefore, the generation area of the waves can be regarded as a sound point source, and the sound waves can be regarded as spherical waves. So, the area of acoustic wavefront is:

(5) $$S=4\pi r^{2}\;$$

Combining Equations (4) and (5), the equation of sound pressure is obtained:

(6) $$p=\frac{\sqrt{pcW/4\pi }}{r}\;$$

where *r* is the distance between the wavefront and the sound source. As is seen in Equation (6), the sound intensity is inversely proportional to the distance between the sensor and the sound source.

## 3. Single Algorithms to Be Fused

### 3.1. TDOA Location Algorithm

The TDOA location algorithm locates the signal source according to the time difference between the signal arriving at different observation points from the signal source and the signal propagation speed to obtain the distance difference between the signal source and different observation points [25,26]. Then, the difference between two distances from the leak hole to two adjacent sensors is used to make rotating hyperboloids, such that a pair of adjacent sensors act as the focal points, and each distance difference is a major axis. Then, the intersection point of the rotating hyperboloids can be calculated according to other mathematical methods (such as the least squares method or Newton iteration method), which is the leak hole position.

Let us assume that the coordinates of sensors A, B, C and D in the planar sensor array are $A(x_{1},y_{1},z_{1})$, $B(x_{2},y_{2},z_{2})$, $C(x_{3},y_{3},z_{3})$ and $D(x_{4},y_{4},z_{4})$, respectively, and the time taken for the signal to reach each sensor is $t_{1}$, $t_{2}$, $t_{3}$ and $t_{4}$, respectively, the time differences between the arrival of the signal at the sensors are $\Delta t_{12}$, $\Delta t_{23}$ and $\Delta t_{34}$ (subscript 1 corresponds to sensor A, 2 corresponds to the sensor B and so on).

According to the ordinates of the sensors, the time differences, the positioning equations, which is also the equations of the rotating hyperboloid, are as follows:

(7) $$\left\{ \begin{array}{l} \sqrt{{\left(x-x_{1}\right)}^{2}+{\left(y-y_{1}\right)}^{2}+{\left(z-z_{1}\right)}^{2}}-\sqrt{{\left(x-x_{2}\right)}^{2}+{\left(y-y_{2}\right)}^{2}+{\left(z-z_{2}\right)}^{2}}\;=\;c(\Delta t_{12}+\xi_{12})\\ \sqrt{{\left(x-x_{2}\right)}^{2}+{\left(y-y_{2}\right)}^{2}+{\left(z-z_{2}\right)}^{2}}-\sqrt{{\left(x-x_{3}\right)}^{2}+{\left(y-y_{3}\right)}^{2}+{\left(z-z_{3}\right)}^{2}}\;=\;c(\Delta t_{23}+\xi_{23})\\ \sqrt{{\left(x-x_{3}\right)}^{2}+{\left(y-y_{3}\right)}^{2}+{\left(z-z_{3}\right)}^{2}}-\sqrt{{\left(x-x_{4}\right)}^{2}+{\left(y-y_{4}\right)}^{2}+{\left(z-z_{4}\right)}^{2}}\;=\;c(\Delta t_{34}+\xi_{34}) \end{array}\right.\;$$

where $\xi_{ij}$ represents the calculated error of the arrival time difference. When these hyperbolic equations are combined with other mathematical estimation methods, such as the least squares method or the Newton iteration method, their approximate solutions can be acquired and when combined provide the leak hole position. Because the Newton iteration method has the advantage of fast convergence and is widely used to solve for the roots of complex equations or equations, we used it in our study.

The iterative formula for estimating the location of leak holes by using the Newton iteration method is as follows:

(8) $$\left[\begin{array}{c} x_{k+1}\\ y_{k+1}\\ z_{k+1} \end{array}\right]=\left[\begin{array}{c} x_{k}\\ y_{k}\\ z_{k} \end{array}\right]-\left\{{\left[f^{\prime }\left(x,y,z\right)\right]}^{-1}\left[\begin{array}{c} f_{1}(x,y,z)\\ f_{2}(x,y,z)\\ f_{3}(x,y,z) \end{array}\right]\right\}|{}_{\begin{array}{c} x=x_{k}\\ y=y_{k}\\ z=z_{k} \end{array}}\;$$

where *f*_1_(*x*,*y*,*z*), *f*_2_(*x*,*y*,*z*), *f*_3_(*x*,*y*,*z*) denotes the difference between the distance difference and the product of time difference and the sound velocity. Supposed that the distances between the sensors and the leak hole is *S*_1_, *S*_2_, *S*_3_, *S*_4_, *f*_1_(*x*,*y*,*z*), *f*_2_(*x*,*y*,*z*), *f*_3_(*x*,*y*,*z*) are:

(9) $$\left\{ \begin{array}{l} f_{1}(x,y,z)=S_{1}-S_{2}-c(\Delta t_{12}+\xi_{12})\\ f_{2}(x,y,z)=S_{2}-S_{3}-c(\Delta t_{23}+\xi_{23})\\ f_{3}(x,y,z)=S_{3}-S_{4}-c(\Delta t_{34}+\xi_{34}) \end{array}\right.\;$$

Here ${\left[\begin{array}{ccc} x_{k} & y_{k} & z_{k} \end{array}\right]}^{T}$ represents the result of the *k*-th iteration, $f^{\prime }(x,y,z)$ is the Jacobian matrix of the system of Equation (9). According to the chosen initial values, the iterative calculations are performed with Equation (8). When the difference between two successive calculations is less than a fixed value, the iteration is stopped, and the final result is obtained. The chosen initial values are key to determining whether the Newton iteration method can converge to the expected value. For a nonlinear equation or system of equations with multiple roots, the iteration will converge to the root closest to the initial value, that is, a local extremum. However, the extreme value may not be a globally optimal solution, and if the initial estimate is not in the lower convex neighborhood of the extreme value, it may cause iterative divergence. Therefore, it is difficult but crucial to correctly choose the initial values of the Newton iteration method, which increases the difficulty in effectively using the TDOA location algorithm [27,28].

**Figure 3:**
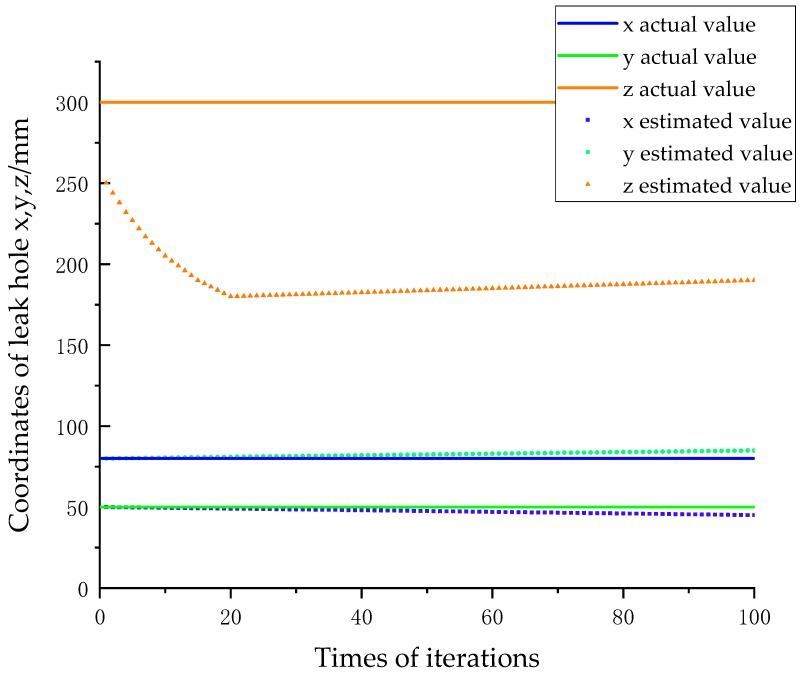
Simulation results of the TDOA algorithm.

Moreover, because the TDOA location algorithm mainly obtains the time difference by calculating the phase difference between the two signals, when the test distance is too large, the phase difference cannot be exactly θ or θ + 360° × n (where n is an arbitrary natural number), and the error of the time difference is unstable. Figure 3 shows the iterative process of using the Newton iteration method to calculate the location of a leak when the leak is located at (−80, 50, 300), and the error of the arrival time difference is within 5 μs. It can be seen that the state of the system converges to a local extremum during the iteration, which is very different from the actual location of the leak.

When the leak is located at (−80, 50, 300), the initial estimated position of the Newton iteration method is (0, 0, 0) becacuse of the lack of leak hole information. When the initial position is far away from the leak position, the iteration will fail to reach the real coordinates of the leak hole only with the detected time differences, and the location error will be extremely high. Therefore, a rough location section is added in the TDOA location algorithm to estimate the initial estimated position of the Newton iteration method.

**Figure 4:**
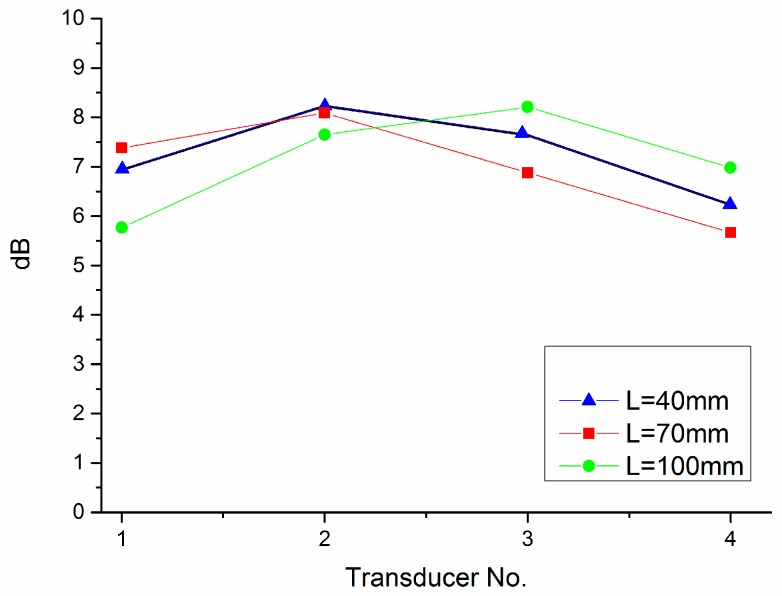
Acoustic intensity of sensor array.

Figure 4 shows the typical acoustic intensity of sensor array, because of the decay characteristics of the ultrasonic waves [15], the sensors are closer to the leak hole than other sensors when the received sound pressure value is higher. For example, in Figure 4, the leak hole is between sensors No. 2 and No. 3. When the detection distance is long, the broken line in Figure 4 is flat, and the acoustic intensity difference of sensors is too little to calculate the leak hole accurately. However, the acoustic intensity can be used to locate the leak position roughly, choose the sensors that are close to the leak hole, narrow the range of leak to two or three sensors, and then estimate the initial estimated position of the Newton iteration method, make the initial position closer to the leak hole, ensure the accuracy and stability of the TDOA location algorithm.

### 3.2. ED Location Algorithm

The principle of the ED location algorithm is to obtain the ratio of the sound pressures at different observation points by analyzing the signal measured by the sensors. These are then converted into ratios of distances from the sound source to different observation points, and according to these distance ratios, the spherical equation group that determines the position of the sound source is obtained. Then, through this method together with other mathematical methods (such as least squares or maximum likelihood estimation), the sound source location is solved.

The sensor array is composed of piezoelectric ultrasonic sensors that are sensitive to sound pressure, and the output value of the sensors is proportional to the sound pressure of the sound wave, so that:

(10) $$u_{i}=g_{i}p_{i}$$

where *i* = 1, 2, 3, 4…, is the number of sensors, and *g_i_* represents the receiving coefficient of sensor *i*.

As is discussed in Section 2.2, the sound intensity is inversely proportional to the distance between the sensor and the sound source. If there is no environmental noise, the sound pressure ratio of sensors *i* and *j* is:

(11) $$k_{ij}=\frac{p_{i}}{p_{j}}=\frac{u_{i}/g_{i}}{u_{j}/g_{j}}=\frac{\left|r-r_{j}\right|}{\left|r-r_{i}\right|}$$

where *r*, *r_i_*, *r_j_* denotes the positional vector of the sound source and sensors: $r={\left[\begin{array}{ccc} x & y & z \end{array}\right]}^{T}$, $r_{i}={\left[\begin{array}{ccc} x_{i} & y_{i} & z_{i} \end{array}\right]}^{T}$, and $r_{j}={\left[\begin{array}{ccc} x_{j} & y_{j} & z_{j} \end{array}\right]}^{T}$.

Therefore, the location equations obtained by the signals measured by the sensors in the planar array are:

(12) $$\left\{ \begin{array}{l} \frac{\sqrt{{\left(x-x_{2}\right)}^{2}+{\left(y-y_{2}\right)}^{2}+{\left(z-z_{2}\right)}^{2}}}{\sqrt{{\left(x-x_{1}\right)}^{2}+{\left(y-y_{1}\right)}^{2}+{\left(z-z_{1}\right)}^{2}}}\;=\;(k_{12}+\zeta_{12})\\ \frac{\sqrt{{\left(x-x_{3}\right)}^{2}+{\left(y-y_{3}\right)}^{2}+{\left(z-z_{3}\right)}^{2}}}{\sqrt{{\left(x-x_{2}\right)}^{2}+{\left(y-y_{2}\right)}^{2}+{\left(z-z_{2}\right)}^{2}}}\;=\;(k_{23}+\zeta_{23})\\ \frac{\sqrt{{\left(x-x_{4}\right)}^{2}+{\left(y-y_{4}\right)}^{2}+{\left(z-z_{4}\right)}^{2}}}{\sqrt{{\left(x-x_{3}\right)}^{2}+{\left(y-y_{3}\right)}^{2}+{\left(z-z_{3}\right)}^{2}}}\;=\;(k_{34}+\zeta_{34}) \end{array}\right.$$

The limitation of the ED location algorithm is mainly reflected in the fact that when the distance between the sensor array and the leak hole is large, the sound pressure ratio is close to 1, which makes this method extremely sensitive to errors and results in reduced positional accuracy.

**Figure 5:**
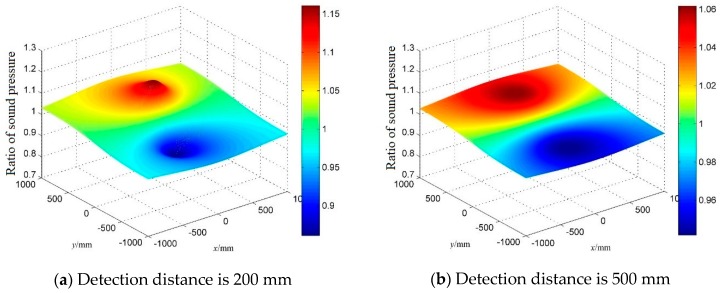
Ideal sound pressure ratio at different detection distances.

Figure 5 shows the ideal sound pressure ratios when the detection distance is 200 mm (a) and 500 mm (b). The coordinates of the two sensors are, respectively, (0,−30,0) and (0,30,0), where the *x* and *y* coordinates of the leak holes are shown on the *x* and *y* axes, and *z* = *d*. As can be seen from Figure 5, as the detection distance increases, or the distance between the leak hole and the midpoint of the two sensors decreases, the sound pressure ratio gets closer to 1. When the coordinates of the leak hole are (10,10,500), and the signal-to-noise ratio of the ultrasonic signal is 30 dB, the measured sound pressure ratio is 0.9683, which is close to the ideal ratio 0.9976. However, the error defined by the Euclidean distance between the estimated location and the true location is 200 mm. It can be seen that the location method is extremely sensitive to errors, which results in low accuracy.

The limitation of the ED location algorithm is also reflected by the time delay of the sensors. Because the time delay between two ultrasonic transducers is unknown, it cannot be guaranteed that the corresponding part of the sampling time of the output signals of the two adjacent sensors can be sampled at the same time, thus resulting in a larger measurement error in the sound pressure ratio used to locate the leak hole.

## 4. Data Fusion Location Algorithm

To address the individual limitations of the TDOA and ED location algorithms, we propose a location algorithm based on data fusion of the feature layer to calculate the location of the leak. Data fusion is a method of improving the final result, or the accuracy of the estimation, by combining multi-sensor information or the final or intermediate results obtained by a variety of algorithms. Data fusion can be divided into three levels: data-level fusion, feature-level fusion and decision-level fusion. The proposed location algorithm is based on the integration of the two aforementioned algorithms, which cannot be fused at the data level. In addition, owing to the large errors of the estimated results from the TODA and ED location algorithms, it is not suitable to use decision-level fusion. Therefore, feature-level fusion is adopted to extract and fuse the eigenvectors of the two algorithms, i.e., the arrival distance difference from the TODA location algorithm and the ratio of the arrival distance from the ED location algorithm, to locate the leak hole.

The common methods of data fusion include probability theory, D–S evidence theory, neural networks and Kalman filtering. This paper adopts the Kalman filtering method, which is suitable for real-time processing of information in dynamic environments.

Kalman filtering is a recursive filter that is commonly used in dynamic systems that contain noise to estimate system state values. However, Kalman filtering theory can be applied only to linear systems. The measurement equation in this paper involves the ratio of the distance from the leak hole to the sensors, which is a nonlinear component. Therefore, the extended Kalman filtering (EKF) method is used for data fusion in this paper. The EKF approach is to linearize the nonlinear system and then perform Kalman filtering. Specifically, the nonlinear state transition equation and the observation equation are expanded by using the Taylor series, wherein the high-order terms are ignored to realize local linearization. The disadvantage of the EKF method is that when the nonlinear state transfer equation and the observation equation are strongly nonlinear, the local linearization will result in large errors. The performance of the EKF method depends on the degree of nonlinearity of the system equations. However, the EKF theory is still widely used because of its simplicity [29,30].

**Figure 6:**
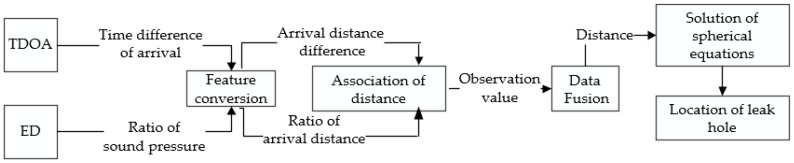
Structure diagram of the proposed location algorithm.

A block diagram of the algorithm’s structure is shown in Figure 6. The TDOA algorithm and the ED algorithm are considered as two subsystems, and the time arrival differences and ratios of sound pressures are obtained. The feature extraction in Figure 6 indicates that the time difference of arrival and the ratio of sound pressure are converted into the arrival distance difference and the ratio of arrival distances, which are combined as observation values for the data fusion. The data fusion part adopts the extended Kalman filter theory to iteratively calculate the location of the leak hole and finally obtain the estimated result.

Let us assume that the *x*, *y* and *z* position coordinates of the leak hole are the system state, so that $r={\left[\begin{array}{ccc} x & y & z \end{array}\right]}^{T}$ is the state vector of the leak location system, and the arrival distance difference and the ratio of arrival distances are the observation values of the system. The data fusion is aimed at acquiring an estimate of the system’s state.

The system’s state equation and observation equation of the leak location system are:

(13) r(k+1)=Φr(k)+ω(k) 

(14) y(k)=h(r(k))+υ(k)

where h(r(k)) is the nonlinear observation function of the system.

(15) $$h(r(k))={\left[\begin{array}{cccccc} s_{1}(k)-s_{2}(k) & s_{2}(k)-s_{3}(k) & s_{3}(k)-s_{4}(k) & \frac{s_{1}(k)}{s_{2}(k)} & \frac{s_{2}(k)}{s_{3}(k)} & \frac{s_{3}(k)}{s_{4}(k)} \end{array}\right]}^{T}\;$$

where $s_{i}(k)$ (*i* = 1, 2, 3, 4) represents the distance between the leak point and sensor *i*.

According to the EKF theory, the a priori estimate of the system and its covariance matrix are:

(16) $$\hat{r}(k+1|k)=\Phi \hat{r}(k|k)\;$$

(17) $$P(k+1|k)=\Phi P(k|k)\Phi^{T}+Q\;$$

The *a posteriori* estimate of the system and its covariance matrix are:

(18) $$\hat{r}(k+1|k+1)=\hat{r}(k+1|k)+K(k+1)\chi (k+1)\;$$

(19) $$P(k+1|k+1)=[I_{3}-K(k+1)H(k+1)]P(k+1|k)\;$$

Among them, the Kalman gain and innovation are:

(20) $$K(k+1)=P(k+1|k)H^{T}(k+1){\left[H(k+1)P(k+1|k)H^{T}(k+1)+R\right]}^{-1}\;$$

(21) $$\chi (k+1)=y(k+1)-h(\hat{r}(k+1|k))\;$$

For the case in which the leak hole is stationary with respect to the sensor array, it is necessary to set a stop condition for the iterative process of data fusion, wherein the iteration ceases when the stop condition is satisfied, and the system state at that time is the calculated result. In this paper, the iterative stopping condition is satisfied when the square of the modulus of the difference between the adjacent two system states is less than a certain fixed value.

**Figure 7:**
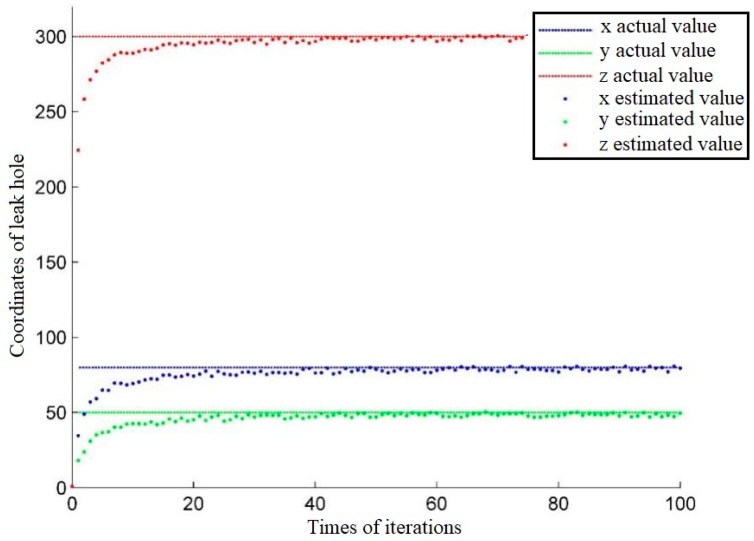
The iterative process of the system state.

Figure 7 shows the iterative process of determining the system state obtained by simulation. The leak hole is located at S(80,50,300), and the sensors are at A(−30,30,0), B(30,30,0), C(30,−30,0) and D(−30,−30,0). From Figure 7, we can see that the system state continuously approaches the real value from the initial estimate, thus demonstrating the validity of the proposed data fusion algorithm.

## 5. Results and Discussion

### 5.1. Design of the Sensor Array

Ultrasonic sensor arrays that are widely used include linear sensor arrays, planar sensor arrays and ring sensor arrays. Among them, linear sensor arrays have the characteristics of simple installation and few calculations; however, they are mainly used for two-dimensional leak location. A ring sensor array is arranged in the shape of a ring, and the detection direction of all the sensors is directed towards the center. The ring sensor arrays are suitable for detecting leaks only in a cylindrical container or a pipe, and they are applicable for only two-dimensional leak location. Therefore, to realize three-dimensional leak location, we used a planar sensor array in our proposed method. The planar sensor array is arranged in a plane, but all the sensors are not collinear. Such an arrangement can be used for three-dimensional location of leak holes, although the amount of calculations performed by the location algorithm is large.

As discussed above, the TDOA location algorithm is based on the difference between distances from the leak hole and the sensors, whereas the ED location algorithm is based on the sound pressure ratio of the sound waves at the sensors. Four sensors result in three pairs of non-correlated sensors that can be used to determine three mutually independent distance differences and sound pressure ratios, which then determine three non-redundant curved surface (rotating hyperboloid, spherical) equations. According to three-dimensional geometry theory, these three surfaces can be used to determine a point. Therefore, the minimum number of sensors to form a planar array and achieve leak location is four.

**Figure 8:**
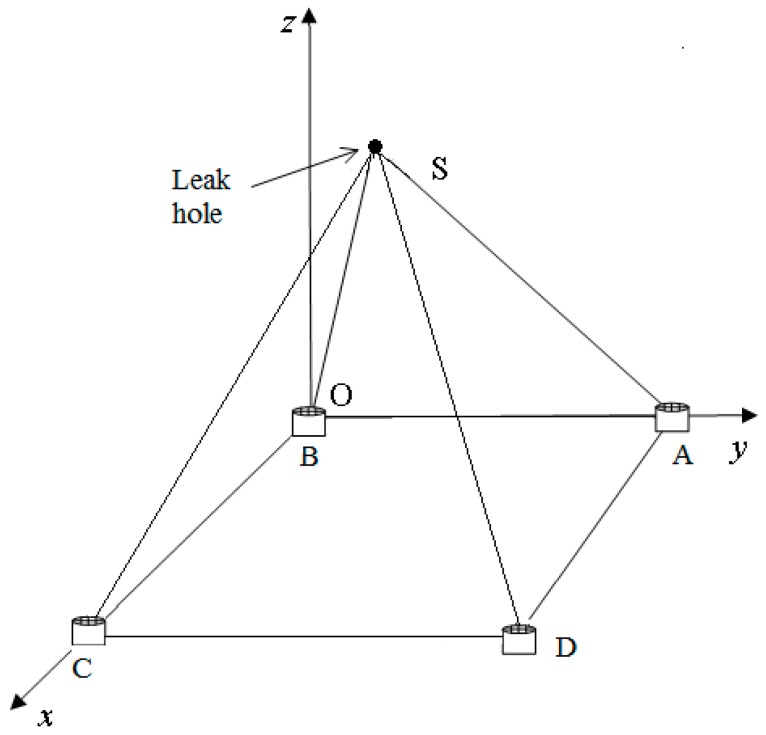
Model of the planar sensor array.

When the number of sensors increases, the accuracy of leak location will be slightly higher. However, when the number of sensors increases to five, the accuracy will be increased by 1.2~1.5%, when the number of sensors increases to six, the accuracy will be increased by 1.3~1.8%, i.e., the accuracy increase is slow while the increase in computational complexity is large. Therefore, we use four sensors to form a square structure, which exhibits advantages of satisfying accuracy, less calculating time and lower cost. A model of the designed sensor array is shown in Figure 8, where S is the leak hole and A, B, C, D are sensors that make up a square sensor array.

### 5.2. Design of the Experimental Device

**Figure 9:**
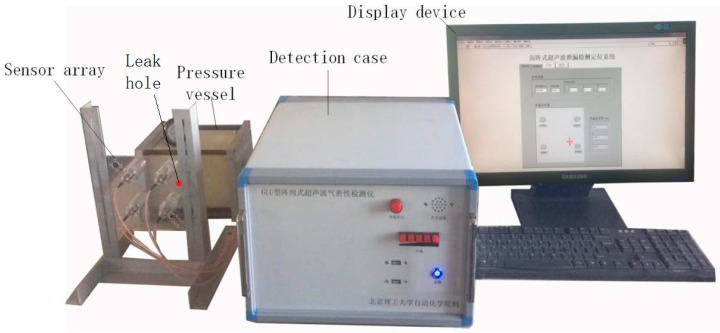
Experimental setup.

To verify the performance of the leak location algorithm proposed in this paper, an experimental device with a four-sensor planar array and a nylon tank with a leak hole of 0.1 mm diameter was set up, as shown in Figure 9. During the experiment, the relative pressure of air in the nylon can was 100 kPa. The four sensors in the experimental device were arranged in a regular quadrilateral structure with a quadrangular side length of 60 mm.

By taking the center of the square sensor array as the origin, the directions perpendicular to the two adjacent sides of the square as the *x* and *y* axes, the direction perpendicular to the sensor array plane and passing through the origin as the *z* axis, a three-dimensional coordinate system was established. The coordinates of the sensors are A(−30,30,0), B(30,30,0), C(30,−30,0) and D(−30,−30,0) (in mm).

The detection case contained an embedded computer, an ultrasonic sensor array, a signal conditioning circuit and a data acquisition card, all of which formed the data acquisition system. On the basis of our sound pressure accuracy requirements, we chose the FUS40-CR sensor manufactured by Fuji Ceramics Corporation (Shizuoka-ken, Japan) in the sensor array because of its small size, light weight and high sensitivity, the basic performance parameters of which are listed below Table 2.

The working process of the acquisition system is as follows: when a leak hole appears and generates an ultrasonic wave, the sensor array will send a signal to the signal conditioning circuit, where the environmental noise is eliminated, and the signal is amplified. Then, the signal is acquired by the data acquisition card (PL2346B manufactured by ZTIC Co., Beijing, China), which can achieve 24 channel data acquisition synchronously and uses a PCI104+ as a data transmission interface, with a sampling rate of 400 kHz. The digital signal is processed by the embedded computer (EPIC-QM77), which uses a 12 V power supply and can support data transmission protocols such as serial ports, USB ports and the PCI104+ interface. The directivity of the sensor is 50 degree. During the detection, the sensor plate needs scan along the tank when the angle between the leak point and the direction of the sensors is beyond this range. The location results are shown on the computer screen.

### 5.3. Experimental Results and Discussion

In the experiments, a leak hole was poked on one side of the gas tank, this side was fixed at 300 mm, 500 mm and 800 mm from the sensor plane. The coordinates of the leak hole are shown in Table 3. Then, we used the TDOA location algorithm, ED location algorithm and data fusion algorithm to estimate the location of the leak hole.

First, the TDOA location algorithm was used. When using the Newton iteration method to calculate the location of the leak, the initial estimate of the leak location coordinates was determined by a rough location based on the sound energy. The error of the calculated leak location is defined as the square root of the sum of difference between the result and the actual leak location, as:

(22) $$e=\sqrt{{\left(x_{e}-x_{S}\right)}^{2}+{\left(y_{e}-y_{S}\right)}^{2}+{\left(z_{e}-z_{S}\right)}^{2}}\;$$

The error defined by Equation (22) indicates the Euclidean distance in three-dimensional space between the calculated result and the actual location of the leak hole. According to Equation (22), the error of TDOA algorithm is shown in Table 4, when using TDOA location algorithm, the location results are shown in Table 4, which shows that after a rough location, the error is stable but still not satisfying when the requirement of accuracy is high, also, the computational burden is increased.

The calculated results and their associated errors of the ED location algorithm are shown in Table 5. As is seen in Table 5, with the ED location algorithm, the results are stable but not accurate enough, because of the unknown delay between two adjacent sensors. The maximum error in the calculated Euclidean distance was 213 mm.

According to the data fusion location algorithm proposed in this paper, the location of the leak hole was calculated. The results and their associated errors and relative errors based on Euclidean distance of the real value are shown in Table 6. As is shown in Table 6, the error of the proposed method can meet the detection requirements, and the error of the calculated Euclidean distance was always less than 20 mm. Therefore, the accuracy and validity of the location algorithm based on data fusion are verified.

There are limitations in distance between sensors and a leak hole. As the distance between the leak hole and sensors increases, the energy of the received signal and the signal-to-noise ratio declines, the accuracy of location will be influenced by the environmental noise. Under condition that the pressure of air in the measured vessel is 300 kPa, the leak hole is 0.1 mm, the signal-to-noise ratio is higher than 3 dB and the leak signal is nearly concealed by the environmental noise when the detection distance is longer than 5m.

The sampling frequency is 400 kHz, thus the smallest time difference change that can be detected is 2.5 μs. When the time difference changes by 2.5 μs, with a sound velocity of 346 m/s, the spacing of ultrasonic sensors is 60mm and the detection distance is 500 mm, the corresponding distance difference change, that is the resolution, is about 2 mm.

**Figure 10:**
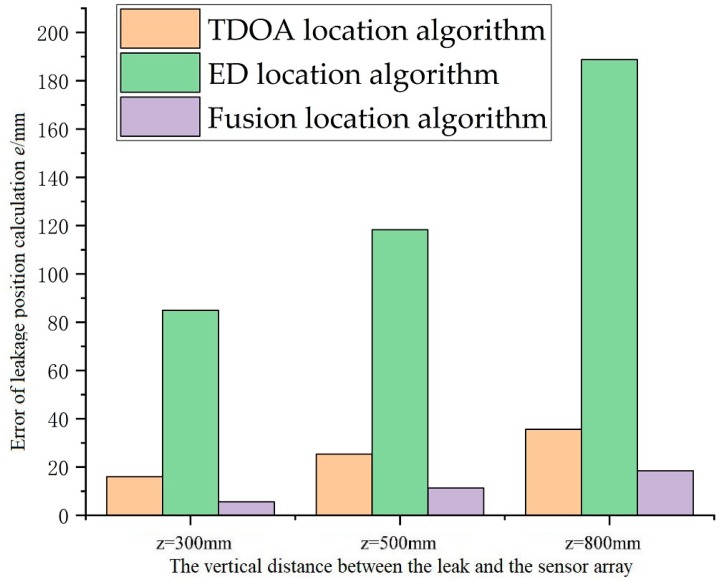
Errors in the calculated results of the three leak position algorithms.

The errors of the TDOA location algorithm, the ED location algorithm and the location algorithm based on data fusion are compared in Figure 10. The location estimation accuracy of the leak hole based on the data fusion location algorithm is higher than that of the TDOA location algorithm by almost a factor of 2 and the ED location algorithm by almost a factor of 10. Moreover, the error increases as the vertical distance from the leak hole to the sensor array plane increases. The main reason for this is that as the distance increases, the signal amplitude and the signal-to-noise ratio decrease.

According to the above experimental results, the method proposed in this paper can estimate the location of the leak hole anywhere within the probed three-dimensional space, and the error is always less than 20 mm, the proposed method has reduced the errors of TDOA and ED location algorithm which are commonly used before. Also, compared with the leak detectors on the market, which will be relatively accurate if the errors are less than 100 mm, and considering the detected vessel, which is on the order of 2 m, an error of 20 mm is accurate enough for the maintenance teams to change or repair the leak vessels, therefore, the proposed method meets practical detection accuracy requirements. These experiments verified the feasibility and effectiveness of the proposed algorithm based on multi-algorithm data fusion.

## 6. Conclusions

In this paper, with the aim of detecting and locating gas leaks by using ultrasonic waves, a location algorithm derived from the data fusion method is proposed, wherein an ultrasonic leak location system based on a planar sensor array was designed and developed. The innovation of this work is that a four element planar array was used to locate leak holes whose locations were estimated by the eigenvectors of the two algorithms (TDOA & ED), and data fusion was applied to the leak hole location. The experiments showed that, under conditions in which the pressure in the measured container was 100 kPa, and the leak hole-sensor distance was 800 mm, the error of the calculated results based on the data fusion location algorithm was always less than 20 mm.

However, there are some limitations of this method. First, the location system is influenced by environmental noise, and it is difficult to detect gas leaks when the leak signal is very small. As a result, the amplifying and filtering circuit should be improved. In addition, we are planning to increase the number of sensors on the sensor array and to study how the layout of the sensor array affects the location results as a way to further improve the accuracy of the location system.

## Figures and Tables

**Table 1:** Comparison of several commonly used leak detection methods.

**Methods**	Differential pressure methods	Mass spectrometry methods	Infrared thermal imaging methods	Ultrasonic methods
**Principle**	Pressure changes in containers	Tracking detection of leakage by tracer gas	Thermal imaging and infrared image processing	Ultrasonic waves produced by leakage
**Advantages**	Low manufacturing costs, simple principle and device	Fast, intuitive and can measure the size of the leak hole directly	High sensitivity, safe operation and fast response	Convenient operation, high sensitivity, low manufacturing costs and accurate positioning
**Defects**	Low accuracy, complicated operation, susceptible to environmental noise	High manufacturing costs, sensitivity is reduced when the pressure in the measured container is variable	High manufacturing costs, susceptible to environmental noise	Susceptible to environmental noise, hard to measure the size of the leak hole

**Table 2:** Basic performance parameters of the FUS40-CR sensor.

Rated frequency	40 kHz
Receiving sensitivity	−46 dB (0 dB = 1 V/Pa)
Bandwidth	6 kHz (−54 dB)
Directivity	50 deg
Range	0.2~6 m
Resolution	9 mm

**Table 3:** Coordinates of the leak hole.

	Leak Positions	S_1_	S_2_	S_3_	S_4_	S_5_	S_6_	S_7_	S_8_	S_9_
Coordinates (mm)										
*x*		−10	80	20	−10	80	20	−10	80	20
*y*		70	50	−20	70	50	−20	70	50	−20
*z*		300	300	300	500	500	500	800	800	800

**Table 4:** Results of the TDOA location algorithm.

Leak Positions		S_1_	S_2_	S_3_	S_4_	S_5_	S_6_	S_7_	S_8_	S_9_
Results (mm)	*x*	−15.4	72.1	28.9	−0.4	30.2	−29.3	30.2	35.6	35.6
	*y*	65.2	65.7	−14.5	87.9	14.1	42.6	38.6	−5.7	−5.7
	*z*	309.7	287.4	290.5	479.2	165.3	278.4	376.3	827.2	827.2
Absolute errors (mm)		12.10	21.63	14.13	29.07	30.28	16.64	35.68	36.77	34.46

**Table 5:** Results of the ED location algorithm.

Leak Positions		S_1_	S_2_	S_3_	S_4_	S_5_	S_6_	S_7_	S_8_	S_9_
Results (mm)	*x*	10.1	110.3	28.9	−47.4	30.2	67.2	−74.3	123.0	53.2
	*y*	30.7	78.5	−14.5	108.3	11.3	45.8	6.2	93.3	48.4
	*z*	370.4	357.7	290.5	411.2	598.4	607.3	606.8	643.6	631.5
Absolute errors (mm)		83.09	71.13	100.60	103.69	116.88	134.43	213.38	167.88	184.86

**Table 6:** Results of the data fusion algorithm.

Leak Positions		S_1_	S_2_	S_3_	S_4_	S_5_	S_6_	S_7_	S_8_	S_9_
Results (mm)	*x*	−12.1	82.9	23.6	−13.3	74.7	26.6	−15.6	87.0	24.5
	*y*	68.5	53.2	−17.7	74.9	44.5	−23.9	65.3	56.5	−17.3
	*z*	305.8	298.9	295.8	511.5	492.2	508.7	817.8	784.6	818.1
Absolute errors (mm)		6.35	4.46	5.99	13.01	10.92	10.12	19.24	17.27	18.85
